# Unmasking Aspergillosis: A Rare Cause of Facial Palsy and Vision Loss in an Immunocompetent Patient—A Case Report and Literature Review

**DOI:** 10.1002/ccr3.70152

**Published:** 2025-02-05

**Authors:** Abdur Rehman, Hafiz Anzal Farooqi, Imran Khan, Fatima Akram, Basit Ali Khan, Arham Ihtesham, Shahzaib Maqbool, Javed Iqbal

**Affiliations:** ^1^ Rawalpindi Medical University Rawalpindi Punjab Pakistan; ^2^ Department of Neurosurgery, Rawalpindi Teaching Hospital Rawalpindi Medical University Rawalpindi Punjab Pakistan; ^3^ PGY 2 NYH+Hospitals/Woodlhul New York New York USA; ^4^ Nursing Department, Communicable Diseases Center Hamad Medical Corporation Doha Qatar

**Keywords:** *Aspergillus*, facial palsy, sphenoid sinus, vision loss, voriconazole

## Abstract

We present a case report of a 45‐year‐old female with acute onset right‐sided facial weakness, worsening vision, and diplopia. Upon evaluation, the patient exhibited total right‐side facial palsy (House‐Brackmann grade IV) with III, IV, and VI Cranial Nerve Ophthalmoplegia. There was no perception of light associated with optic atrophy on ocular examination. Computed tomography and magnetic resonance imaging showed a sizeable sphenoid sinus mass compressing the right optic nerve, consistent with neoplasia or invasive fungal infection. Eventually, we removed the mass endoscopically through a transnasal approach and diagnosed it as invasive Aspergillosis on histopathological examination, even though no previous record of immunodeficiency existed. The patient received intravenous followed by oral voriconazole. Postoperatively, there was partial recovery of her facial nerve function. This case highlights the importance of considering invasive fungal infections, even in immunocompetent patients, when managing space‐occupying lesions with neuro‐ophthalmic involvement.

## Graphical abstract

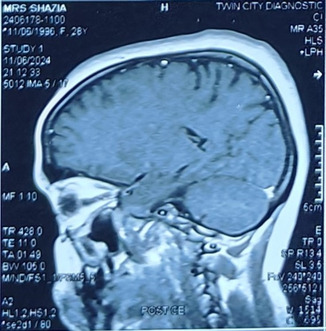

**Summary**

- Invasive Aspergillosis of the sphenoid sinus, though rare in immunocompetent patients, can cause severe cranial neuropathies and vision loss.
- Prompt diagnosis and surgical intervention, followed by antifungal therapy, are critical to preventing irreversible complications in such atypical presentations.

## 1 Introduction

Invasive Aspergillosis of paranasal sinuses is commonly observed as a fungal infection among patients with immune deficiency. Studies have found that inherited immunodeficiency or the use of immunosuppressive drugs (e.g., steroids) associates with invasive fungal sinusitis [1]. Invasive Aspergillosis of the sphenoid sinus is one of the manifestations of immunodeficiency, causing local destruction and complications in surrounding structures and requiring urgent surgical interventions to minimize poor outcomes [2]. The diagnosis of invasive sino‐orbital fungal infections remains challenging due to their rare presentation in immunocompetent individuals and their overlap with other orbital pathologies. Imaging modalities such as contrast‐enhanced computed tomography (CT) or magnetic resonance imaging (MRI) are critical for detecting sinus and orbital involvement, with MRI offering better visualization of soft tissue inflammation, orbital apex, and cavernous sinus involvement. Histopathological confirmation, often using fungal stains such as Gomori methenamine silver (GMS), is essential for definitive diagnosis. Non‐invasive techniques like fine‐needle aspiration cytology can also aid diagnosis in select cases [3].

Management typically involves a combination of surgical and medical therapy. Surgical debridement or more extensive procedures such as exenteration are often necessary to remove infected tissues, especially in cases with posterior orbital or intracranial extension. First‐line antifungal therapy includes itraconazole or amphotericin B, with the latter being associated with significant side effects that may limit its use. Voriconazole is emerging as an effective alternative but is cost‐prohibitive for many patients [3]. Early intervention is critical, as untreated cases have poor outcomes, including high mortality rates due to intracranial spread [3].

The presence of invasive fungal infection in immunocompetent individuals is a rare occurrence. In a study by Dabas et al. (2022), an approximate incidence of 1.4% of invasive fungal disease affecting the sinuses was noted, of which those who were immunocompetent were an even smaller number [4]. Herein, we are reporting a rare occurrence of invasive Aspergillosis of the Sphenoid sinus in a 45‐year‐old female with no known immunodeficiency, posing a diagnostic and therapeutic challenge.

## 2 Case Presentation

We report a 45‐year‐old female patient admitted to our neurosurgery ward with the symptoms of acute right facial weakness and progressive vision loss in the right eye. Over 3 days, this presented insidiously, with the patient noticing facial asymmetry that morning along with the inability to close her right eyelid and drooping at the right angle of her mouth. She also complained of double vision and progressively blurring of the vision in her right eye. This included episodic, self‐resolving vertigo. She reported no headache, fever, known infection, nasal congestion, discharge, or sinus pain. No trauma or neurological deficits were part of her history. Nevertheless, baseline investigations were done; they were all within the reference range. These included a complete blood count (CBC), liver function tests, renal function tests, and serum electrolytes. The only abnormality was a raised CRP × (1.25 mg/dL, ref.:< 1 mg/dL) × and ESR × (30 mm/h, ref.:< 20 mm/h).

On examination, the patient was awake and oriented but exhibited House‐Brackmann grade IV, complete right‐sided facial palsy. She was noted to have profound facial nerve dysfunction with a complete inability to close her right eye, elevate her eyebrow, or move the right side of her mouth. There was a loss of landmark in the nasolabial fold on the affected side. The lack of light perception in the right eye on examination indicated severe optic nerve damage. On further examination, there was the restriction of the right eye with limitation in all directions suggestive of severe palsy involving cranial nerves III (oculomotor), IV (trochlear), and VI (abducens). The pupillary reflexes to light were poorly preserved on the right, while the fundoscopy showed features of optic atrophy.

## 3 Methods

At the onset of this case, possible differential diagnoses considered included idiopathic orbital inflammatory syndrome, bacterial orbital cellulitis, rhinosinusitis, and orbital neoplasms such as lymphoma or metastasis. Other conditions like Tolosa‐Hunt syndrome, optic neuritis, or fungal infections such as mucormycosis and sino‐orbital aspergillosis were also part of the diagnostic considerations due to overlapping clinical features. These differential diagnoses guided the initial clinical and imaging workup, as well as the decision to pursue histopathological confirmation.

**FIGURE 1:**
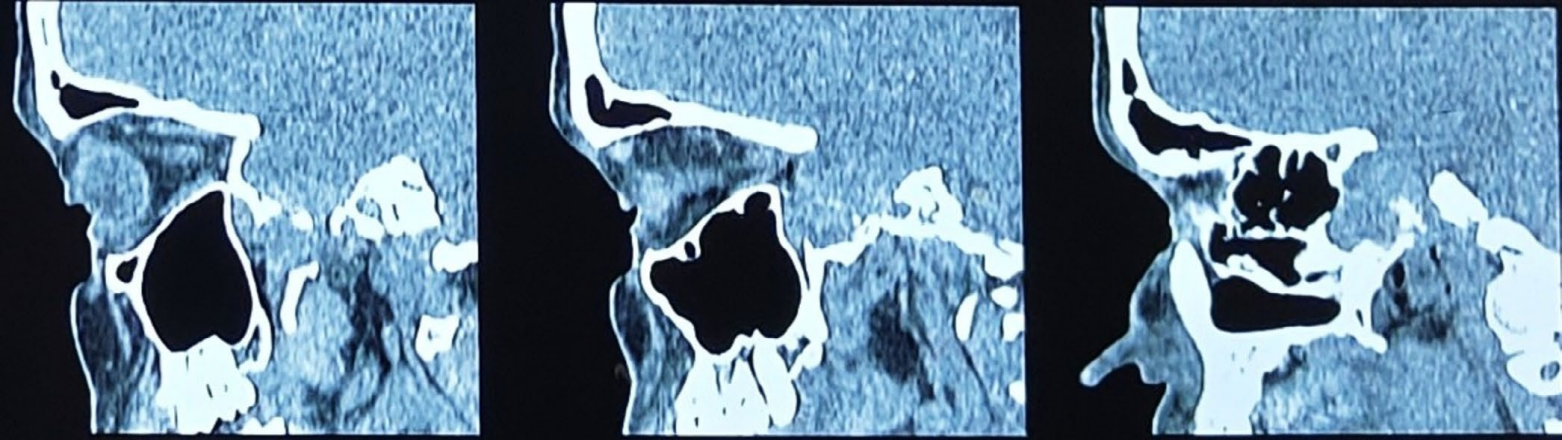
Sagittal computed tomography: Sphenoid sinus mass can be seen.

**FIGURE 2:**
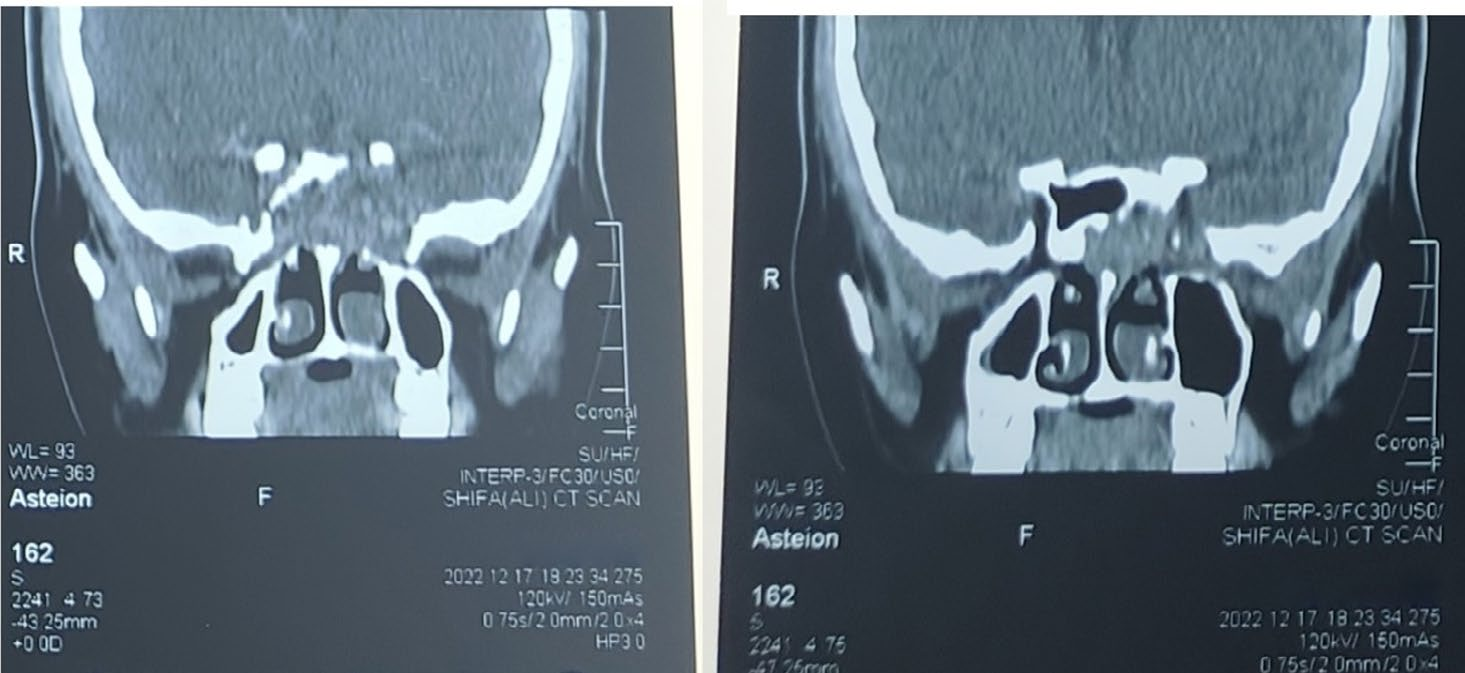
Coronal computed tomography: Bony erosion surrounding sphenoid sinus mass.

Given her progressive and incapacitating symptomatology, urgent neuroimaging was done. A CT of the brain and sinuses showed a substantial space‐occupying lesion of the sphenoid sinus extending into the sellar area with significant compression of the right optic nerve by the mass, as shown in Figures 1 and 2. However, there was no intracranial extension or involvement of other cranial structures. A CT angiogram was done to rule out a glomus tumor, and it was normal.

**FIGURE 3:**
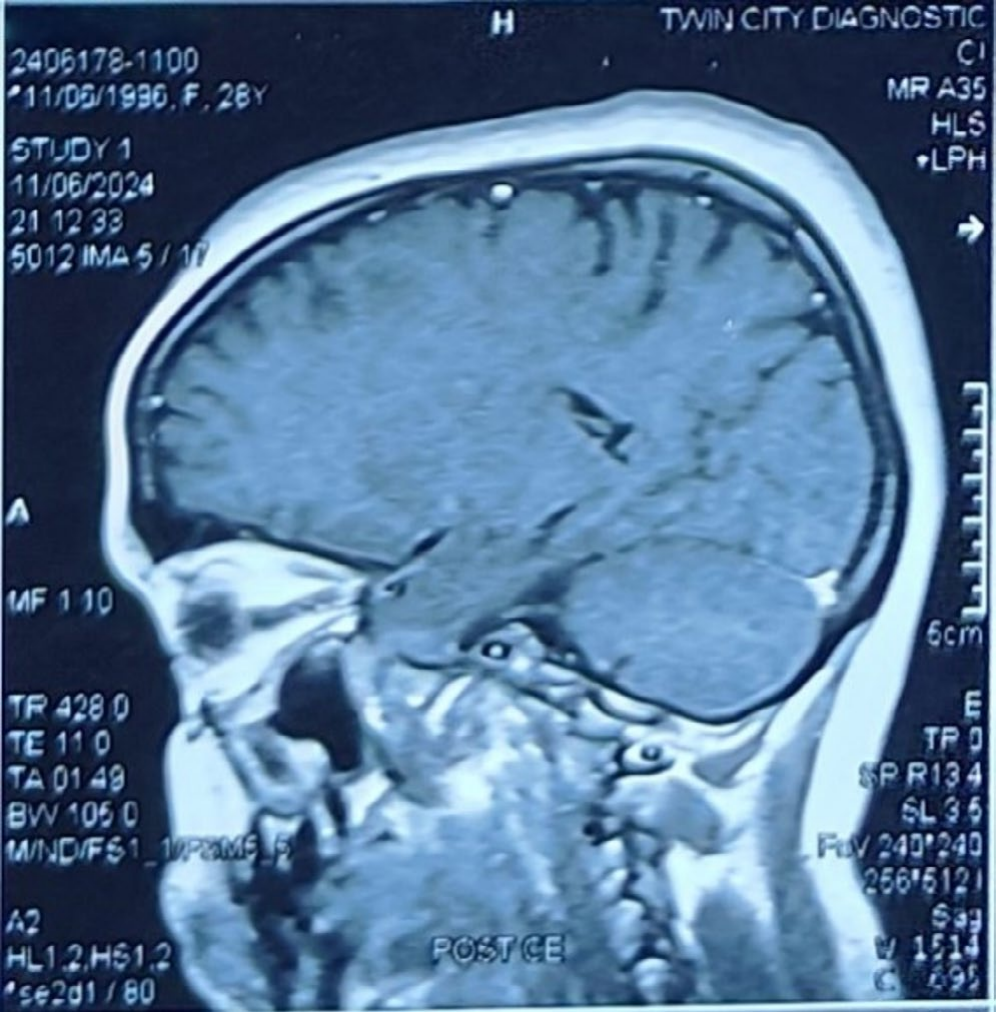
Magnetic resonance imaging: Sphenoid sinus lesion compressing the optic nerve.

**FIGURE 4:**
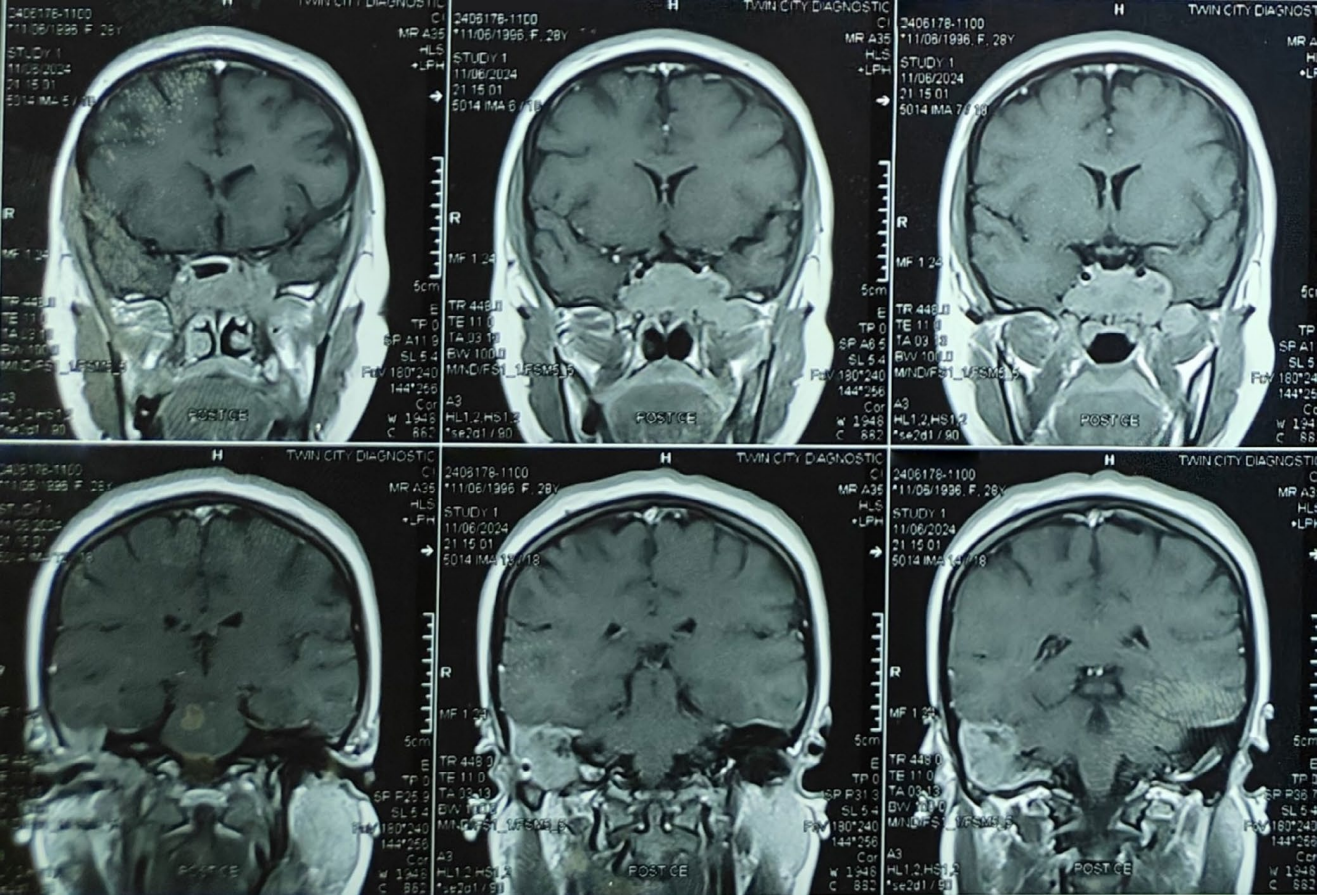
Magnetic resonance imaging: Invasion of sphenoid mass into adjacent cranial structures.

**FIGURE 5:**
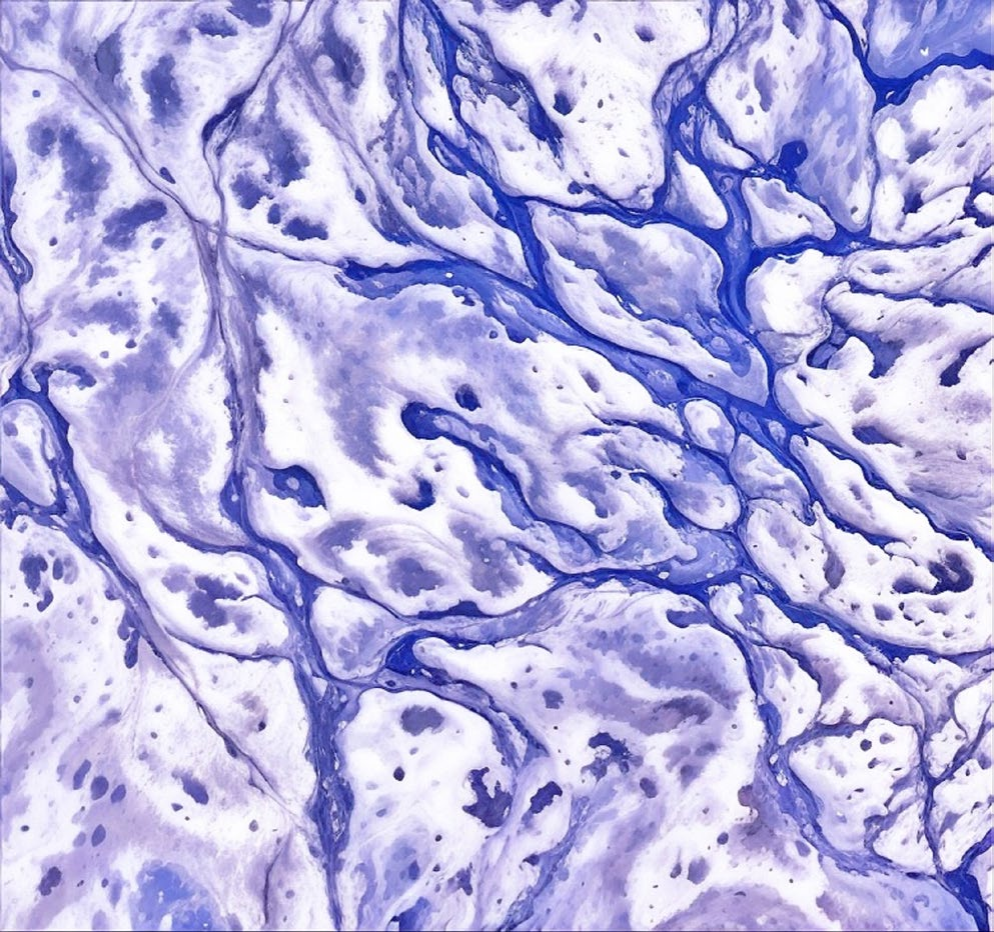
Histopathology: *Aspergillus* hyphae with acute‐angle branching.

Following this, an MRI of the soft tissues' characterization was done, detailing the invasive mass with evident marked pressure on the optic nerve, encroaching upon the adjacent cranial nerves, as shown in Figures 3 and 4. The radiological findings indicated that the mass may be neoplastic, and a pituitary adenoma or meningioma may thus be considered. A pituitary profile ruled out an adenoma, showing all values within normal limits. The profile included assessments of serum prolactin, growth hormone, cortisol, adrenocorticotropic hormone, thyroid‐stimulating hormone, free thyroxine, luteinizing hormone, and follicle‐stimulating hormone, providing a comprehensive evaluation of pituitary function. The suspicion of invasive fungal infection arose as a possible differential diagnosis, especially in the sinuses' involvement pattern and erosion in the surrounding bone. Considering the possibility of potential risks of loss of vision and neurological complications, endoscopic transnasal resection of the sphenoid sinus lesion was performed on the patient. Preoperative endoscopic examination revealed a firm, pale‐yellow to brown mass with necrotic areas. The surface appeared irregular, with evidence of friability and mild bleeding on contact. Signs of invasion into the surrounding bony structures were evident. The team sent a specimen of the mass for histopathological confirmation of invasive Aspergillosis. Histopathological examination of the specimen confirmed invasive Aspergillosis. The fungal hyphae were identified using GMS and periodic acid‐Schiff stains, which revealed septate hyphae with acute‐angle branching, a hallmark of *Aspergillus* species. The hyphae exhibited uniform diameter and invaded the surrounding necrotic tissue, confirming angioinvasion. These findings were pivotal in establishing the diagnosis, particularly given the atypical presentation in an immunocompetent patient, as shown in Figure 5. It is an important diagnosis to make, as invasive Aspergillosis usually occurs in immunocompromised patients; this patient had no known immunodeficiency. The laboratory further investigated underlying immunocompromised factors, including HIV status, HbA1c, and a CBC; all results were unremarkable.

## 4 Conclusion and Results

Thus, she was postoperatively started on intravenous antifungals with voriconazole, which was the drug of choice in invasive Aspergillosis (two loading doses of 6 mg/kg in the first 24 h, followed by 4 mg/kg twice daily for 10 days). She tolerated the surgery well, with good postoperative recovery. Patient was discharged on 200 mg twice daily dose of oral voriconazole for 6 months. Her right facial weakness gradually improved, and her right facial nerve function partially recovered over subsequent weeks. Her vision in the right eye never recovered, and damage to the optic nerve has been considered irreversible. At the six‐month follow‐up, partial recovery of facial nerve function was seen: the patient could partially close her right eyelid, and some motility of the lower half of the face had returned. Generally, the facial asymmetry still existed, but overall, her facial palsy had improved to House‐Brackmann grade III.

This report highlighted a rare case of invasive aspergillosis of Sphenoid sinus, which was observed in an immunocompetent individual with neurological involvement leading to facial nerve palsy. Histological analysis, followed by surgical debridement and voriconazole remain the primary management approach for such patients. This case report underscores that timely and effective therapeutic intervention is crucial in treating invasive fungal infection.

## 5 Discussion

This case represents one of the few instances of invasive *Aspergillus* involving the sphenoid sinus in an immunocompetent patient, highlighting the diagnostic and therapeutic challenges posed by such rare presentations. Invasive Aspergillosis is typically observed in immunocompromised states such as organ transplantation, hematological malignancies, or uncontrolled diabetes [5]. However, in rare cases, it can occur in immunocompetent hosts, necessitating a high index of suspicion to avoid delays in diagnosis and treatment. Early diagnosis is essential to prevent catastrophic outcomes, such as permanent vision loss or neurological impairments, as demonstrated in this case [6]. Literature supports the critical need for prompt intervention in such scenarios to mitigate irreversible complications [7].

The presentation was atypical because this is a case in which all the common symptoms of sinusitis (headache, nasal congestion, and sinus pain) are absent, yet cranial neuropathies showed up. Such an unusual presentation may result in delayed diagnosis with increased risks of irreversible damage, such as permanent loss of vision in this patient's right eye [8]. The multiplicity of the cranial nerves involved and the rapidity with which the symptoms had progressed pointed toward its invasive nature. Neuroimaging data, particularly MRI, were critical in differentiating this lesion from alternative options, such as pituitary adenoma or meningioma, which may also present radiologically similarly [9]. In the same way, the role of non‐contrast CT scans is slightly limited in invasive fungal sinusitis cases compared to noninvasive forms. The contrast‐enhanced CT scan is used preferentially to delineate the anatomy of surrounding vessels [10].

Surgical debridement followed by antifungal therapy remains the primary treatment for invasive fungal sinusitis [11]. As a result, in this case, the sphenoid mass was removed endoscopically through the nose, followed by antifungal therapy with voriconazole, the preferred medicine for invasive Aspergillosis. Voriconazole was more effective than amphotericin B in treating invasive Aspergillosis [12, 13]. Thus, early initiation is essential to improving outcomes. The delayed intervention led to extensive optic nerve involvement, a well‐known complication in cases of invasive Aspergillosis of the sphenoid sinus. Despite aggressive management, the patient did not regain her vision in the right eye. Therefore, the postoperative recovery of this patient shows partial improvement in the function of the facial nerve and thus points to the fact that early surgical intervention can prevent deterioration in cranial nerve function. However, the prognosis for full recovery of cranial nerve impairments in invasive Aspergillosis is often poor, especially if the optic nerve is involved.

This case report demonstrates the need for a high level of suspicion for invasive fungal infection, even in immunocompetent patients with unusual characteristics. Despite normal laboratory results, potential underlying factors contributing to immunocompromise should be considered. Undiagnosed conditions such as subtle primary immunodeficiencies, diabetes, or autoimmune disorders may impair the immune response without overt clinical symptoms. Medications like corticosteroids or non‐steroidal anti‐inflammatory drugs, taken recently or intermittently, might transiently suppress immunity [3]. Additionally, environmental exposures, including mold or organic dust, may elevate the risk of invasive fungal infections in susceptible individuals [1]. These considerations underscore the complexity of diagnosing and managing invasive Aspergillosis, even in apparently immunocompetent patients [11].

Early surgical intervention, combined with the administration of suitable antifungals, is required to prevent irreversible problems, as evidenced by the patient's partial return of cranial nerve function.

## Author Contributions

**Abdur Rehman:** conceptualization, formal analysis, investigation, resources, supervision. **Hafiz Anzal Farooqi:** data curation, methodology, visualization. **Imran Khan:** methodology, project administration, writing – original draft. **Fatima Akram:** data curation, investigation, methodology, writing – original draft. **Basit Ali Khan:** data curation, writing – original draft, writing – review and editing. **Arham Ihtesham:** data curation, software, writing – original draft. **Shahzaib Maqbool:** software, writing – review and editing. **Javed Iqbal:** funding acquisition, software, supervision, writing – review and editing.

## Ethics Statement

The need for ethical approval was waived by the Ethics review board of our institution.

## Consent

Written informed consent was obtained from the patient for collection and publication of case details.

## Conflicts of Interest

The authors declare no conflicts of interest.

## Permission to Reproduce Material From Other Sources

N/A.

## Data Availability

Data is available from authors upon reasonable request.
